# The efficacy of artificial intelligence in urology: a detailed analysis of kidney stone-related queries

**DOI:** 10.1007/s00345-024-04847-z

**Published:** 2024-03-14

**Authors:** Gökhan Cil, Kazim Dogan

**Affiliations:** 1 Department of Urology, Bagcilar Training and Research Hospital, University of Health Sciences, Istanbul, Turkey; 2 Department of Urology, Faculty of Medicine, Istinye University, Istanbul, Turkey

**Keywords:** Artificial intelligence, ChatGPT, Urology, Kidney stones

## Abstract

**Purpose:**

The study aimed to assess the efficacy of OpenAI's advanced AI model, ChatGPT, in diagnosing urological conditions, focusing on kidney stones.

**Materials and methods:**

A set of 90 structured questions, compliant with EAU Guidelines 2023, was curated by seasoned urologists for this investigation. We evaluated ChatGPT's performance based on the accuracy and completeness of its responses to two types of questions [binary (true/false) and descriptive (multiple-choice)], stratified into difficulty levels: easy, moderate, and complex. Furthermore, we analyzed the model's learning and adaptability capacity by reassessing the initially incorrect responses after a 2 week interval.

**Results:**

The model demonstrated commendable accuracy, correctly answering 80% of binary questions (*n*:45) and 93.3% of descriptive questions (*n*:45). The model's performance showed no significant variation across different question difficulty levels, with *p*-values of 0.548 for accuracy and 0.417 for completeness, respectively. Upon reassessment of initially 12 incorrect responses (9 binary to 3 descriptive) after two weeks, ChatGPT's accuracy showed substantial improvement. The mean accuracy score significantly increased from 1.58 ± 0.51 to 2.83 ± 0.93 (*p* = 0.004), underlining the model's ability to learn and adapt over time.

**Conclusion:**

These findings highlight the potential of ChatGPT in urological diagnostics, but also underscore areas requiring enhancement, especially in the completeness of responses to complex queries. The study endorses AI's incorporation into healthcare, while advocating for prudence and professional supervision in its application.

## Introduction

Artificial Intelligence (AI) and data learning technologies have continued to break new ground, rapidly transforming the landscape of various industries, with healthcare [1, 2]. A compelling manifestation of this progression is the emergence of advanced language models such as OpenAI's ChatGPT (Generative Pretrained Transformer), which has demonstrated promising potential in diverse fields [3, 4]. In medicine, its innovative applications are making substantial contributions, particularly in patient care and recordkeeping [5–8].

Creating systems with ChatGPT can enhance patient self-management of health conditions [9]. By utilizing the capabilities of ChatGPT, healthcare professionals can automate the documentation process of patient interactions and medical histories, thus ensuring a more efficient and streamlined medical records system [10]. By inputting dictated notes, healthcare providers can use ChatGPT to summarize significant aspects like symptoms, diagnoses, and treatments and extract pertinent data from patient records such as laboratory or radiological reports [8, 11–13]. Despite the escalating significance of AI in healthcare [14], there remains a lack of comprehensive studies investigating its real-world application in diagnostics [15].

ChatGPT's capabilities extend to facilitating patient management [16]. Providing dosage guidelines and vital information regarding potential side effects, drug interactions, and other essential factors is another way ChatGPT can assist urology [17]. As urological diseases often present complex diagnostic challenges, there is a burgeoning interest in evaluating the role and effectiveness of AI tools like ChatGPT in this domain [18]. The burgeoning landscape of AI in urology is fascinating, and ChatGPT's role in it is just beginning [18]. This investigation is crucial for urologists and AI researchers, healthcare providers, and stakeholders involved in the evolving realm of digital health [19, 20]. We can better understand the potential of AI and guide its development to optimize patient outcomes in the complex field of urology.

This research article explores the performance of ChatGPT in diagnosing urological conditions, providing a fresh perspective on the integration of AI in healthcare diagnostics. We will delve into the structure and abilities of ChatGPT, critically analyze its performance in identifying urological diseases, and discuss the potential benefits and limitations of utilizing such a tool in the medical field.

## Materials and methods

### Study design and setting

The present study was conducted in May–June 2023 by two endourologists (GC, KD) who prepared questions containing clear and unequivocal answers about kidney stones. The main requirement was that the questions have clear and undisputed answers based on the established medical guideline—EAU Guidelines 2023. They were tasked to generate a set of 90 specific questions that centered around kidney stones. All procedures followed the Declaration of Helsinki's ethical rules and principles.

### Data collection process

An essential part of our study design was to control for potential biases. To this end, we entrusted a single researcher with inputting all the questions into ChatGPT. This procedure helped maintain consistency in the question-asking process and ensured the AI model received the questions as intended. Following the AI's generation of responses, the physicians who created the questions were given these answers for evaluation.

### ChatGPT model

It is a state-of-the-art language model developed by OpenAI and trained on a diverse range of internet text. It is an AI system that uses machine learning techniques, specifically a variant of the transformer model architecture called GPT. The version as of the time of the study was based on the GPT-4 (limited-premium version-June 2-2023) architecture. As of the last update in September 2021, it cannot access or learn from information post-training, including real-time events, updates, or sources. In the medical field, the model has potential applications such as assisting in patient care, managing medical records, and even acting as a supportive tool for diagnostics, the last of which is the main focus of this study. While the AI can provide valuable assistance and information, a healthcare professional should review and verify all output, as the AI model does not possess medical judgment.

### Application and evaluation procedure

**Fig. 1:**
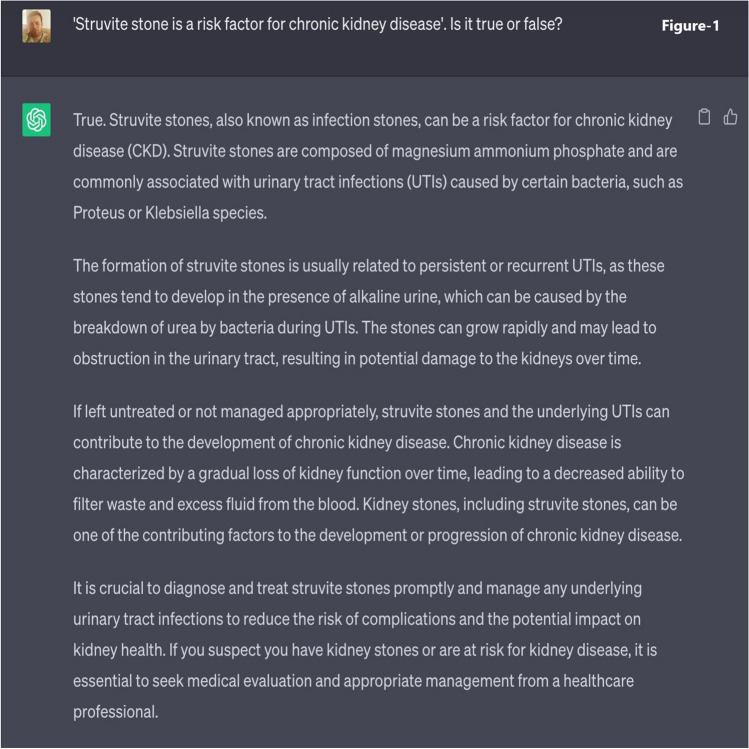
Template of true–false (yes/no) question posed to ChatGPT

**Fig. 2:**
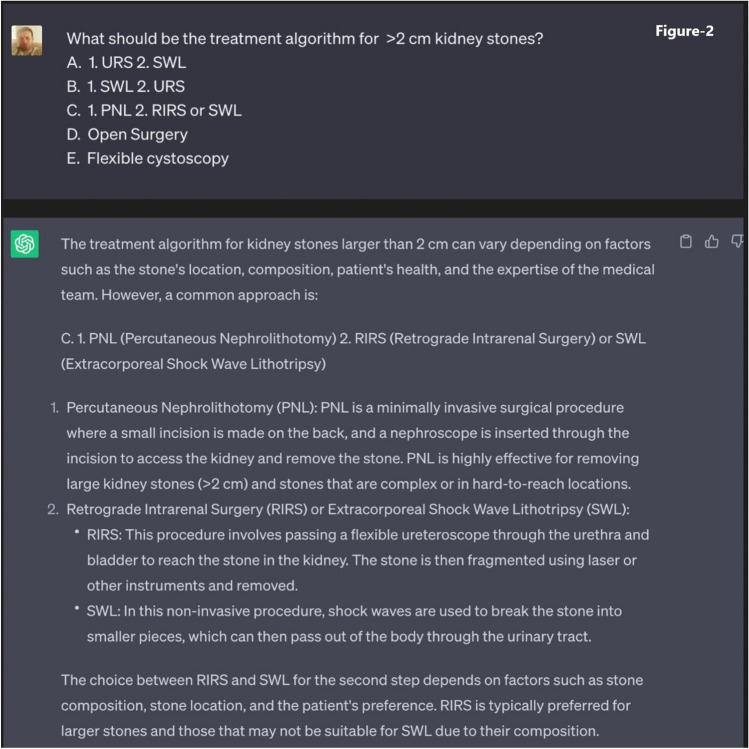
Template of multiple-choice (descriptive) question posed to ChatGPT

The responsibility of each physician was to develop a total of ninety questions. Half of these questions required binary responses (yes/no or true/false), as seen in Fig. 1, while the other half were more descriptive, with the possibility of having multiple correct answers, as seen in Fig. 2. To ensure a varied difficulty level for a more comprehensive evaluation, these questions were classified into three categories: easy, moderate, and complex.

Two primary scales were used to evaluate the responses provided by ChatGPT: an accuracy scale and a completeness scale. Accuracy Rating: we implemented a six-point Likert scale to evaluate the accuracy of each response (1; entirely incorrect, 2; mostly incorrect, 3; equal parts correct and incorrect, 4; more correct than incorrect, 5; almost entirely correct, 6; correct). This detailed scale allowed a more nuanced understanding of the AI model's performance. Completeness Rating: a three-point Likert scale was utilized for evaluating the completeness of each answer (1; incomplete, addresses some aspects of the question but misses significant parts or points, 2; adequate, addresses all aspects of the question and provides the necessary minimum information, 3; comprehensive, addresses all aspects of the question and provides additional information or context beyond expectations).

An integral part of our study was reevaluating answers initially deemed incorrect by ChatGPT (those scoring less than three on the accuracy scale). We considered it essential to gauge the impact of time on the AI's accuracy. Accordingly, after a gap of 14 days, the same questions were presented to ChatGPT again. The physicians then reassessed and scored the updated responses.

### Statistical analysis

The statistical analysis conducted in this study was two-fold and was designed to give a comprehensive understanding of the performance of ChatGPT in urological conditions. All collected data were summarized using descriptive statistical methods. These include the median, the middle value when all data points are arranged in ascending order, and the mean, the average of all data points. Given the nature of the data, we chose to use non-parametric tests for inferential statistics. The Mann–Whitney U test was used to compare two independent groups. The Kruskal–Wallis test was employed when more than two groups were compared. The Wilcoxon signed-rank test was used in the follow-up evaluation of the answers. This non-parametric statistical hypothesis test is used when comparing two related samples or repeated measurements on a single sample to assess whether their population mean ranks differ. All tests were two-sided, and a *p*-value of less than 0.05 was considered significant. Analyses were performed using statistical software (SPSS-v26, IBM Co., USA).

## Results

In a set of 90 questions, we find differing levels of accuracy for two types of responses: binary (*n*:45) and descriptive (*n*:45). For the binary responses, we note 9 incorrect answers. Subtracting this from the total, we find 36 correct responses (80%). On the other hand, descriptive responses performed slightly better. Out of 45 questions, only three were marked as incorrect. Doing similar calculations, we found there were 42 correct answers (93.3%).

In the context of binary questions, the performance of the ChatGPT model demonstrated variance based on the complexity of the queries. For questions classified as easy, the model achieved an average accuracy score of 4.7 ± 2, with a median of 6. For questions of moderate difficulty, the mean accuracy score was 4.9 ± 1.8, with the median observed as 6. The model reported an average accuracy score of 4.4 ± 1.6 for the more challenging questions, with a median of 5. Regarding response completeness, differences were apparent across the difficulty levels, with average completeness scores reported as 2.5 ± 0.9, 2.5 ± 0.8, and 2.1 ± 0.9 for easy, moderate, and complex questions, respectively. The *p*-values for accuracy and completeness were 0.548 and 0.417, respectively, suggesting that no significant variations were discernible among different difficulty levels (Table 1).

**Table 1:** Statistical values and *p*-values for variable accuracy and completeness across different difficulty levels

Variables	Easy (*n*:15)	Moderate (*n*:15)	Complex (*n*:15)	*P*-value
Accuracy (*binary*)	4.7 ± 2 (6; 4.1)	4.9 ± 1.8 (6; 3.1)	4.4 ± 1.6 (5; 2.5)	0.548
Accuracy (descriptive)	5.6 ± 0.7 (6; 0.5)	5.3 ± 1.0 (6; 1.1)	4.7 ± 1.5 (5; 2.4)	0.112
Completeness (*binary*)	2.5 ± 0.9 (3; 0.8)	2.5 ± 0.8 (3; 0.7)	2.1 ± 0.9 (2; 0.8)	0.417
Completeness (descriptive)	2.7 ± 0.6 (3; 0.4)	2.7 ± 0.6 (3; 0.4)	2.5 ± 0.7 (3; 0.6)	0.613

Each value can be understood as mean ± standard deviation (median; variance). These values are provided for each variable according to the specified difficulty level (easy, moderate, complex). The analysis was conducted using the Kruskal–Wallis *H* test for comparing groups of difficulty levels, a non-parametric method for testing whether samples originate from the same distribution

**Fig. 3:**
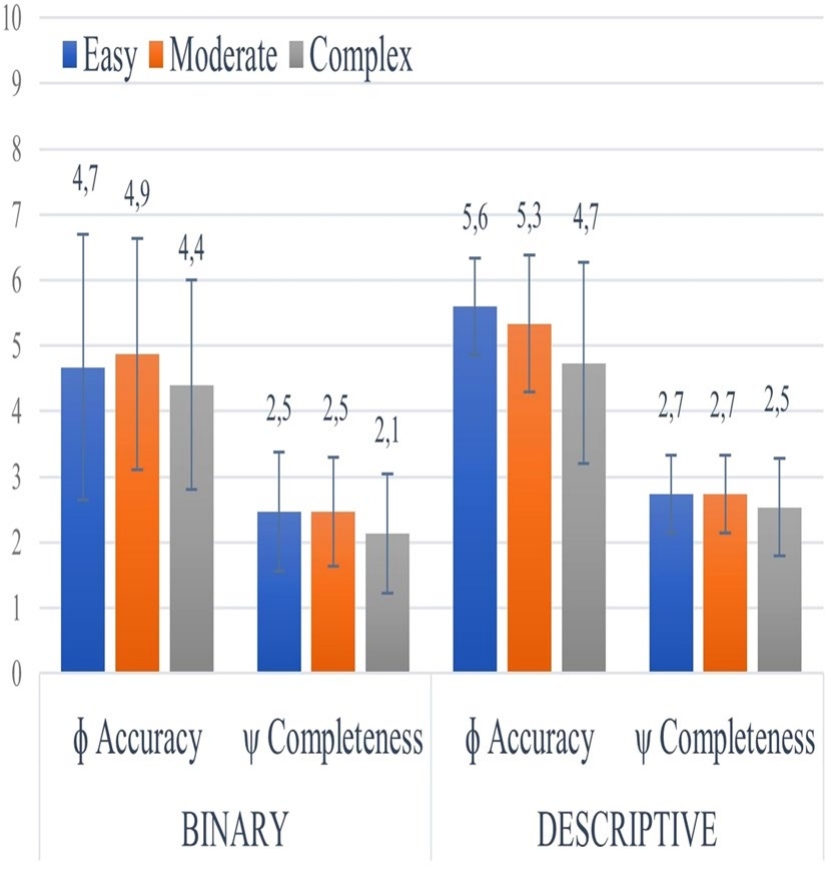
Graph of accuracy and completeness across different difficulty levels

A similar trend was observed regarding descriptive questions, with the model's performance varying according to the difficulty level of the queries (Fig. 3). The model achieved an average accuracy score of 5.6 ± 0.7 for easy questions, with a median of 6. The mean accuracy score for questions of moderate complexity was 5.3 ± 1, with a median of 6. For the complex questions, the average accuracy score was 4.7 ± 1.5, with a median of 5. Regarding the completeness of responses, the average scores for easy, moderate, and complex questions were 2.7 ± 0.6, 2.7 ± 0.6, and 2.5 ± 0.7, respectively. The *p*-values for accuracy and completeness were 0.112 and 0.611, respectively, suggesting the absence of any significant differences among the varying difficulty levels in the accuracy and completeness of responses.

Following 2 weeks, these same questions were asked again to gauge any improvements in the model's accuracy. Notably, in the first batch of 90 questions, the model's accuracy was poor in nine binary and three descriptive questions. A comparison was drawn between the initial and reassessed responses to analyze the model's learning capacity and adaptability over time. These initial poorly-answered 12 questions displayed a mean accuracy of 1.58 ± 0.515. Two weeks later, when these 12 questions were re-asked, the mean accuracy significantly improved to 2.83 ± 0.937 (*p* = 0.004).

In the binary category, 2-week improvements were seen across different difficulty levels. For easy questions, the accuracy scores increased from 1 and 2 in the first assessment to 2 and 3 in the reassessment. For questions with a moderate difficulty level, the model's performance improved even more significantly with rescored values of 2 and 4, compared to initial scores of 1 and 2. The highest difficulty level also demonstrated a consistent enhancement in accuracy. The initial accuracy score was 2, whereas, during reassessment, the scores were 2, 3, and 5, indicating the model's increased understanding and accuracy over time. There was a noticeable improvement in the descriptive question category for 2-weeks. For the moderate questions, the model’s score rose from 2 in the initial assessment to 3 in the follow-up. Questions marked as complex difficulty level initially scored 2 and 1 but increased to 3 and 2 in the reassessment, signifying the model's ability to comprehend these questions better.

## Discussion

The increasing prominence of AI in various fields raises the question of its efficacy in delivering accurate and comprehensive responses, particularly in complex areas like healthcare. The study evaluated the performance of an AI language model, OpenAI's GPT-4, in addressing questions related to urological kidney stones. The investigation focused its performance on binary and descriptive questions of varying difficulty levels, using the parameters of accuracy and completeness. The results offer intriguing insights into the capability of the AI model in processing and responding to complex medical queries.

The application of artificial intelligence, particularly ChatGPT, a natural language processing tool by OpenAI, in medicine, specifically urology, is a rapidly growing field of interest [19]. Several studies have sought to investigate this model's utility, quality, and limitations in providing medical advice and patient information, alongside its role in academic medicine [19]. It is clear from these studies that while ChatGPT does possess considerable potential, significant concerns remain regarding its accuracy, the quality of its content, and the ethical implications of its utilization [8]. Cocci et al. investigated the use of ChatGPT in diagnosing urological conditions, comparing its responses to those provided by a board-certified urologist [21]. It provided appropriate responses for non-oncology conditions, but its performance fell short in oncology and emergency urology cases. Furthermore, the quality of the information provided was deemed poor, underlining the need to evaluate any medical information provided by AI carefully. They found that the appropriateness of ChatGPT's responses in urology was around 52%, significantly lower than our findings of 80% accuracy for binary responses and 93.3% for descriptive responses.

Similarly, studies conducted by Huynh et al. [17] and Deebel et al. [18]. which focused on evaluating the utility of ChatGPT as a tool for education and self-assessment for urology trainees, found it wanting. While there were instances of ChatGPT providing correct responses and reasonable rationales, its overall performance was lackluster, with persistent justifications for incorrect responses potentially leading to misinformation [17, 18]. A similar disparity is seen when we compare our findings to those of Huynh et al., which found that ChatGPT was correct on 26.7% of open-ended and 28.2% of multiple-choice questions [17]. Our study's accuracy rate was substantially higher, showing that ChatGPT may have a more practical application in specific contexts and modes of questioning. Deebel et al. found that ChatGPT's performance improved when dealing with lower-order questions [18], a finding echoed by our results, showing a high accuracy level for both easy and moderate-level questions.

The results of our study echo those of previous research into the accuracy and quality of ChatGPT's responses in the field of urology, albeit with some differences. When compared to previous studies, it is evident that our research has shown a higher degree of accuracy in both binary and descriptive responses [17, 18, 21, 22]. Coskun et al. found that while ChatGPT was able to respond to all prostate cancer-related queries, its responses were less than optimal, often lacking in accuracy and quality [23]. This suggests that reliance on ChatGPT for accurate patient information should be exercised cautiously. However, the study by Coskun et al. reminds us of the limitations of AI-generated patient information, which are also echoed in our study. Although our accuracy scores were higher, the quality and completeness of the responses provided by ChatGPT were lower than desired, highlighting the need for improvements in the model's performance, particularly in the context of more complex or challenging queries.

Based on the results of this scientific study, the performance of the artificial intelligence model in answering questions about urological kidney stones can generally be considered high. The model typically received above-average scores for accuracy and completeness when answering questions of varying difficulty levels. Responses to easy questions typically received high scores in accuracy and completeness, while the accuracy and completeness scores for responses to more challenging questions were slightly lower. However, these scores are generally within acceptable levels. The standard deviations of the accuracy and completeness scores indicate a degree of variability in the model's performance from question to question. Additionally, the results of the Kruskal–Wallis tests suggest that the difficulty level of the questions does not impact the accuracy or completeness of the responses, implying that the model responds to questions of varying levels with similar capabilities.

Despite the valuable insights derived from this study, certain limitations have been acknowledged. Primarily, the specificity of the 90 questions related to urological kidney stones is recognized, a limitation that does not fully capture the extensive range of medical queries the model may encounter. Moreover, the categorization of questions as 'easy,' 'moderate,' or 'complex' is acknowledged to be somewhat subjective and potentially interpreted differently by various healthcare professionals. While the accuracy and completeness of the responses were evaluated, it is noted that other essential factors, such as relevance, coherence, and the ability to interact in real-time clinical context were not considered. It is therefore suggested that future research employ more comprehensive studies, incorporating larger and more diverse datasets as well as additional evaluative parameters, in order to more fully ascertain the capabilities and limitations of the GPT-4 model within a healthcare context.

As a conclusion, ChatGPT model is generally capable of answering questions about urological kidney stones accurately and comprehensively, by showing promising results in terms of its learning capacity and adaptability over time. However, it is important to note that performance does show some variability from question to question, especially when dealing with more complex or challenging questions. These findings highlight areas for learning and improvement, underscoring the importance of continuous training and updates.

## Data Availability

The data that support the findings of this study are available from the corresponding author, upon reasonable request.
